# Nucleation and Growth of Porous MnO_2_ Coatings Prepared on Nickel Foam and Evaluation of Their Electrochemical Performance

**DOI:** 10.3390/ma11050716

**Published:** 2018-05-02

**Authors:** Wenxin Huang, Jun Li, Yunhe Xu

**Affiliations:** School of Materials Engineering, Shanghai University of Engineering Science, Shanghai 201620, China; vensin_huang@163.com (W.H.); zgsdxyh@gmail.com (Y.X.)

**Keywords:** MnO_2_, coating, electrodeposition, nucleation and growth

## Abstract

Porous MnO_2_ was uniformly electrodeposited on nickel foam in MnSO_4_ solution, which was applied as the electrode of supercapacitors. The nucleation/growth mechanisms of porous MnO_2_ were investigated firstly. Then two kinds of electrochemical measuring technologies, corresponding to the cycle voltammetry (CV) and galvanostatic charge-discharge, were adopted to assess the electrochemical performance of MnO_2_ electrodes. The results demonstrated that the deposition of MnO_2_ on nickel foam included four stages. Prior to the deposition, an extremely short incubation period of about 2 s was observed (the first stage). Then the exposed nickel foam was instantly covered by a large number of MnO_2_ crystal nuclei and crystal nuclei connected with each other in a very short time of about 3 s (the second stage). Nucleation predominated in the second stage. The sharply rise of current was caused by the increase in substrate surface area which due to nucleation of MnO_2_. Grain boundaries grew preferentially due to their high energy, accompanied with a honeycomb-like structure with the higher surface area was formed. However, accompanied with the electrochemical reactions gradually diffusion-controlled, the current presented the decline trend with increasing the time (the third stage). When the electrochemical reactions were completely diffusion-controlled, the porous MnO_2_ coating with an approximately constant surface area was formed (the fourth stage). MnO_2_ coatings deposited for different time (30, 60, 120, 300 s) exhibited a similar specific capacitance (CV: about 224 F/g; galvanostatic charge-discharge: about 264 F/g). Comparatively speaking, the value of MnO_2_ deposited for 600 s was highest (CV: 270 F/g; galvanostatic charge-discharge: 400 F/g).

## 1. Introduction

Recently the ever-increasing demands for clean and renewable energy and growing awareness of the environmental protection promote the development of efficient energy storage and energy conversion equipment [1,2,3,4]. Supercapacitors, which are known as energy storage devices have also caused wide attention due to their high energy density, long service life, fast charge/discharge rate capacity and environmental friendliness [5,6]. According to charge storage mechanism, supercapacitors mainly include two types: Faraday pseudocapacitors, in which charge is stored in the surface and bulk of electrodes by Faradaic reactions and electric double layer capacitors, in which charge is stored in the electric double layer [7]. Among the factors affecting the performance of supercapacitors, electrode materials play a crucial role. Thus far, the most widely used electrode materials include carbon [8,9,10], conducting polymers [11,12,13] and metal oxides [14,15,16,17]. Among mental oxides, MnO_2_ is considered a promising candidate for electrode materials due to its extremely high theoretic specific capacitance up to 1380 F/g, low cost and abundant source [18,19,20,21].

Numerous studies have focused on the synthesis of MnO_2_ electrodes by different methods, such as hydrothermal [22,23,24,25], sol-gel [26,27], precipitation [28], electrodeposition [29,30,31,32,33,34,35,36] and other methods [37,38]. Materials are usually prepared powder form. Some materials with high conductivity and binders are added into the powder in suitable ratios. Periodically, a small amount of water or ethanol is added to form the rubber-like mixture. Then the mixture is placed on the substrate to form electrodes by rolling or pressing. The process is complex and may result in poor reproducibility of the electrochemical performance of electrodes. Electrode conductivity also weakens given the addition of additives with poor conductivity [32]. Compared with other methods, electrodeposition can be directly employed to prepare the electrode by MnO_2_ deposition on different substrates. Electrodeposition technique has been widely used to prepare the coatings due to its accurate controllability, ease operation, high deposition rate, good repeatability and low cost [39]. Nirmal Peiris et al. [40] synthesized Mg(OH)_2_ coatings on the surfaces of TiO_2_ by means of electrodeposition at room temperature. The deposition process was explained by the changes in zeta potential of TiO_2_ and Mg(OH)_2_. The zeta potentials of these compounds are closely related to the pH of the electrolyte. The iso-electric points of Mg(OH)_2_ and TiO_2_ total 12 and 6.5, respectively. The substance is positively charged below the iso-electric point but it is negatively charged above the value. At a pH of 6.5–12, the electrostatic interaction between the positively charged Mg(OH)_2_ and negatively charged TiO_2_ promotes the deposition of Mg(OH)_2_ on the surface of TiO_2_.

Fan et al. [29] prepared a MnO_2_-based electrode by electrodepositing a composite consisting of MnO_2_ and polypyrrole on carbon cloth. In the galvanostatic charge-discharge test, the specific capacitance value was 325 F/g when a current density of 0.2 A/g was applied. Mishra et al. [30] synthesized MnO_2_ electrodes on stainless steel foil by potentiostatic, potentiodynamic and galvanostatic modes. The specific capacitance of these electrodes reached a maximum of 325.6 F/g via the potentiostatic mode when a current density of 1 mA/cm^2^ was applied. Xiao et al. [31] prepared amorphous MnO_2_ on nickel foam via electrodeposition. The electrode exhibited a high specific capacitance of 837.6 mF/cm^2^ at 0.5 mA/cm^2^ and an excellent rate capability (84% of that was retained after 2000 cycles). The substrate type has an appreciable effect on the electrochemical performance of the electrode. Comparatively, nickel foam has attracted significant attention due to its high porosity, uniformity, light weight and good electrical conductivity [31,41,42]. The current investigations on MnO_2_ electrodeposition on nickel foam primarily focus on the influence of electrode preparation on the electrochemical performance of electrodes. A limited number of studies reported the nucleation/growth mechanisms of MnO_2_ electrodeposition on nickel foam. However, theoretical studies on nucleation and growth mechanisms remain essential. Such studies offer guidance for obtaining MnO_2_ electrodes with excellent electrochemical performance.

In this study, the nucleation and growth mechanisms of MnO_2_ on nickel foam were revealed by in detail. Morphological evolution of MnO_2_ deposited at different time (3, 7, 20, 40 and 120 s) was analyzed to confirm its nucleation and growth mechanisms in detail. The MnO_2_ electrodes prepared at different time periods (30, 60, 120, 300 and 600 s) were used for electrochemical performance testing. The electrochemical properties of MnO_2_ deposited for different time was evaluated in Na_2_SO_4_ by two kinds of electrochemical measuring technologies (cyclic voltammertry (CV) and galvanostatic charge-discharge).

## 2. Experimental

Nickel foam was selected as the electrode substrate (Shanxi Powder Source Battery Materials Co., Ltd., Taiyuan, China). The substrates with a dimension of 25 mm × 10 mm × 1 mm were immersed in 0.1 M HCl solution for 10 min at room temperature to remove some oxides adhering to nickel foam surfaces. Then, the substrates were ultrasonically cleaned in acetone solution and deionized water. After that, the samples were dried at room temperature and weighted using an electronic balance (0.1 mg, Sartorius BSA124S, Beijing, China).

An electrochemical workstation system (CHI 660E, CH Instruments Inc., Shanghai, China) with a conventional three−electrode system was used to investigate by CV the deposition process of MnO_2_. The test was carried out in a 0.14 M MnSO_4_ solution as the electrolyte at room temperature. Pretreated nickel foam was taken as the working electrode and large pretreated graphite flake with large a dimension of 25 mm × 20 mm × 1 mm was taken as the counter electrode. Those electrodes were soaked in the electrolyte (height 10 mm); the distance between them was controlled at approximately 15 mm. A saturated calomel electrode (SCE) as the reference electrode was used to measure the relative potential applied to the working electrode. Cyclic voltammograms were recorded between −0.2 V and 1.8 V at different scan rates of 5, 10 and 20 mV/s. The potential window was expanded from −0.8 V to 1.8 V to observe the third reduction peak at a scan rate of 20 mV/s. The nucleation and growth mechanisms of MnO_2_ were revealed by chronoamperometry with different applied potentials (1.3 V–2.0 V) for 60 s.

MnO_2_ was deposited at different (0 s to 600 s) at an applied potential of 2.0 V to investigate the morphological evolution with time. A Hitachi SU8010 SEM (Hitachi Limited, Tokyo, Japan) was applied to observe the morphologies of MnO_2_. The product deposited at 120 s was used for analyzing chemical compositions and phase constituents. Chemical compositions were confirmed by an energy dispersive spectrometer (EDS, GENESIS, EDAX Inc., Mahwah, New Jersey, USA). Phase constituents of the product deposited at 120 s were identified by an X-ray diffractometer (XRD, PANalytical X’ Pert Pro, Eindhoven, The Netherlands). Cu Kα radiation with a wavelength of 0.1540560 nm) was used. The chemical states of the deposit were determined by an X-ray photoelectron spectrometer (XPS, Shimadzu/Kratos, Kyoto, Japan), utilizing a monochromatic Al Kα source. Two scan modes corresponding to the survey and XPS spectra were calibrated by the C1s peak located at 284.8 eV.

The electrochemical performances of the MnO_2_ electrodes prepared at different time periods (30, 60, 120, 300 and 600 s) and the nickel foam without MnO_2_ deposition were assessed in a 0.5 M Na_2_SO_4_ electrolyte by two kinds of electrochemical measuring technologies (CV and galvanostatic charge–discharge). Prior the tests, the MnO_2_ electrodes were cleaned for five times in deionized water and dried at room temperature. Then they were weighed. The weight of deposited MnO_2_ was calculated by the difference in weight of nickel foam before and after electrodeposition. The CV measure was performed with the potential ranging from −0.2 V to 0.8 V at a scan rate of 5 mV/s. For the galvanostatic charge–discharge test, the potential was swept from −0.2 V to 0.8 V with a current density of 1 A/g applied. Considering that traces of MnO_2_ were deposited (approximately 6% of nickel foam in weight), measurement errors were possibly produced when weighting of the electrode before and after the depositing MnO_2_. This condition subsequently resulted in calculation errors when obtaining the specific capacitance of the electrode. To minimize errors, nickel foam was weighted thrice before and after deposition. Then, the average values were obtained to calculate the weight of deposited MnO_2_. The electrochemical performance was measured thrice in CV and galvanostatic charge–discharge tests, to obtain excellent reproducibility. The specific capacitance of MnO_2_ was calculated based on the measured electrochemical performance.

## 3. Results and Discussion

### 3.1. Formation Process of MnO_2_

**Figure 1:**
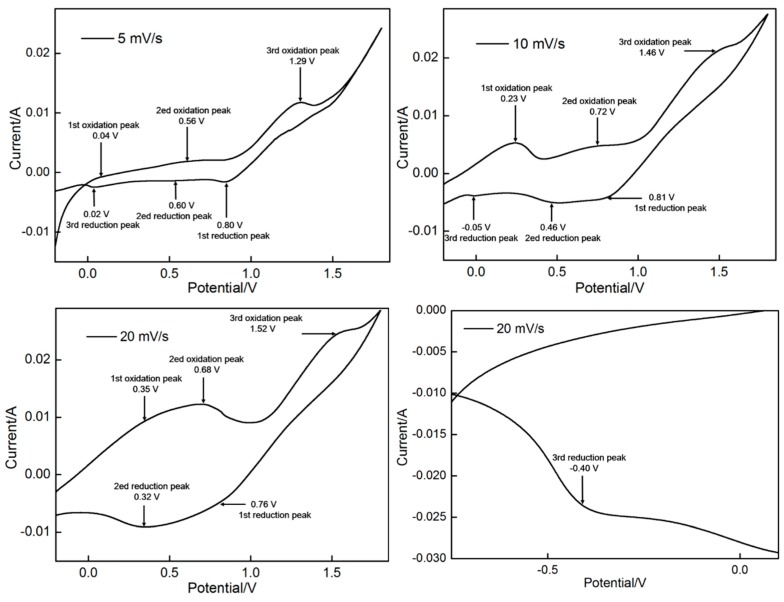
CV curves recorded on foam nickel.

Figure 1 shows three CV curves obtained at different scan rates (5, 10 and 20 mV/s). Three pairs of redox peaks can be observed in the CV curves obtained at 5 and 10 mV/s, indicating that three oxidation reactions occurred with increasing potential. Reduction reactions correspondingly occurred during reverse sweep. At 5 mV/s scanning rate, the peak potentials of the three oxidation peaks reached 0.04, 0.56 and 1.29 V, the matching reduction peak potentials were indexed as 0.02, 0.60 and 0.80 V, respectively. With increasing scanning rate from 5 mV/s to 10 mV/s then finally to 20 mV/s, the oxidation peaks moved toward higher potentials and the corresponding reduction peaks were located at lower potentials (Figure 1). The third reduction peak cannot be observed at 20 mV/s. However, the third peak appeared at −0.4 V when the potential window expanded from −0.8 V to 1.8 V (the fourth image in Figure 1). The evident trend changes should be attributed to the reactions that seriously deviated from the equilibrium state with increasing scanning rate. The first peak located between 0.04 V to 0.35 V should be related to nickel foam oxidation. The other oxidation peaks represented the oxidation of Mn^2+^ to MnO_2_. In general, MnO_2_ can be electrodeposited via two pathways, the predominant of which mainly depends on electrolyte environment. In concentrated acidic electrolytes, MnO_2_ is preferentially synthesized via the disproportionation pathway. On the contrary, MnO_2_ is synthesized via the hydrolysis pathway in less concentrated acidic electrolytes. The reactions in the two pathways can be described as follows [43,44,45].

(1) $${\mathrm{Mn}}^{2+}\to {\mathrm{Mn}}^{3+}+e^{-},$$

Disproportionation:

(2) $$2{\mathrm{Mn}}^{3+}\to {\mathrm{Mn}}^{2+}+{\mathrm{Mn}}^{4+},$$

(3) $${\mathrm{Mn}}^{4+}+2H_{2}O\to {\mathrm{MnO}}_{2}+4H^{+},$$

Hydrolysis:

(4) $${\mathrm{Mn}}^{3+}+2H_{2}O\to \mathrm{MnOOH}+3H^{+},$$

(5) $$\mathrm{MnOOH}\to {\mathrm{MnO}}_{2}+H^{+}+e^{-},$$

The 0.14 M MnSO_4_ is not a strong acidic electrolyte (pH value is 3.2). Thus, the hydrolysis pathway may play a leading role in MnO_2_ synthesis. The second oxidation peak between 0.56 and 0.72 V corresponded to the oxidation of Mn^2+^ to Mn^3+^ (Reaction 1). Then, by hydrolysis reaction (Reaction 4), Mn^3+^ was further converted to porous MnOOH, which is an intermediate product that adheres to nickel foam surfaces. When the potential increased to a range of 1.29 V to 1.52 V, MnOOH was further transformed into MnO_2_ by Reaction 5.

### 3.2. Nucleation and Growth Mechanism

**Figure 2:**
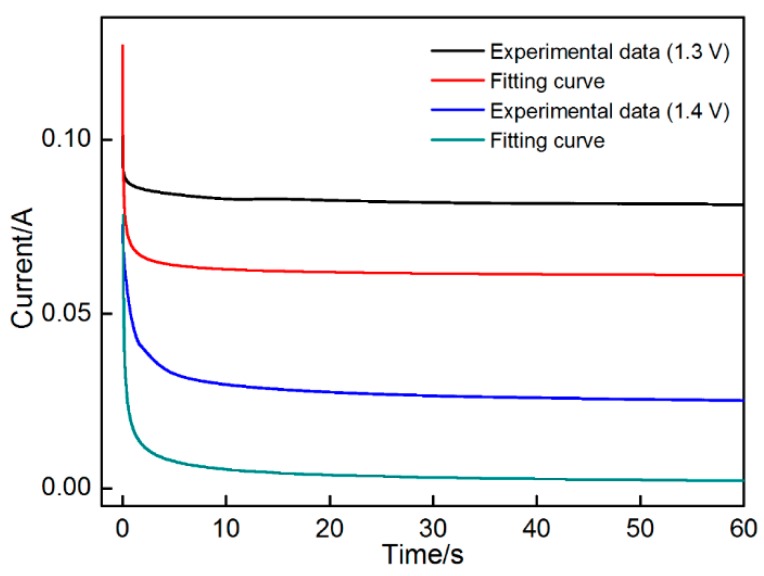
Chronoamperograms obtained on nickel foam.

Chronoamperometry method can be used to reveal the nucleation and growth processes of MnO_2_ by obtaining the current response to different step potentials. Figure 2 shows the chronoamperometry curves obtained by applying different deposition potentials (1.3 V and 1.4 V) on nickel foam in 0.14 M MnSO_4_ solution. A transient current is generated to charge the double layer after application of step potentials. Accompanied with the reactions (Mn^2+^→Mn^3+^ + e^−^), the number of active Mn^2+^ ions around the electrode dropped dramatically. The reactions belong to the diffusion-controlled process, whereas the current correspondingly reduced and finally stabilized. No strong signs indicating MnO_2_ nucleation and growth on nickel foam were observed. However, the CV results indicated that MnO_2_ formed at a potential of approximately 1.3 V. This inconsistency may be attributed to the slight difference between the two potentials. The driving force was insufficient to promote the nucleation and growth of more MnO_2_. Thus, MnO_2_ formation cannot be characterized in the chronoamperometry curves.

The deposition of MnO_2_ can be approximately regarded as a semi-infinite diffusion limited process, under which the Cottrell equation can be applied to establish the relationship between the two variables (current and time) in an ideal chronoamperometry experiment [46]:

(6) $$i_{t}=\frac{nFAD^{1/2}C}{{\left(\pi t\right)}^{1/2}},$$

where *i_t_* refers to the current (*A*), *n* represents the number of electrons gained or lost in the electrochemical reaction, *F* represents the Faraday constant, *A* denotes the electrode surface area immersed in the electrolyte (m^2^), *D* indicates the diffusion coefficient (m^2^/s) and *C* denotes the concentration of Mn^2+^ in the electrolyte (mol/m^3^). According to the Cottrell equation, *n*, *F*, *D* and *C* are all unchanged parameters.

The surface area of electrode (*A*) is the only parameter that significantly changes in this system. When no new phase forms on the electrode surface, *A* also yields a constant value. Thus, the Cottrell equation can be used to fit the data of chronoamperometry curve at the potentials of 1.3 and 1.4 V. The fitting results appear below.

At 1.3 V potential: $i_{t}=0.009t^{-1/2},\;\frac{nFAD^{1/2}C}{\pi }=0.009$

At 1.4 V potential: $i_{t}=0.01734,\;\frac{nFAD^{1/2}C}{\pi }=0.01734$

The two curves cannot coincide with each other (especially at the potential of 1.4 V). With prolonged time, the current measured higher than the theoretical value. Thus, the surface area (*A*) of the electrode may change resulting from nucleation and growth of MnO_2_. The fitting result, as confirmed by the CV test, further proves that MnO_2_ formed at 1.3 and 1.4 V.

**Figure 3:**
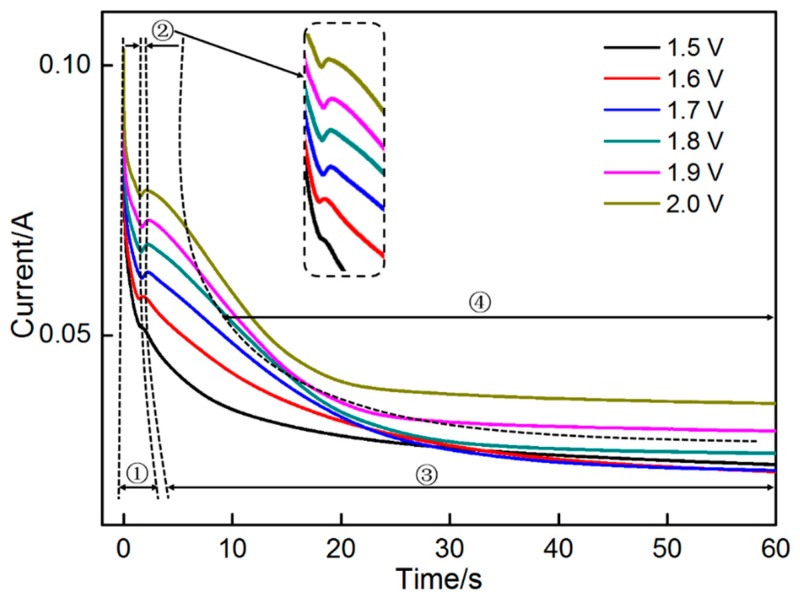
Chronoamperograms obtained on nickel foam.

The curves changed significantly when the potential exceeded 1.4 V (shown in Figure 3), indicating the formation of a large number of MnO_2_. The entire deposition process can be divided into four stages, namely, the nucleation-incubated stage, nucleation-dominated stage, growth-dominated stage and diffusion-controlled stage. When a potential was applied, a large number of active ions migrated rapidly toward the electrode, causing the instantaneous increase in the current. After an extremely short incubation period of approximately 2 s, the chronoamperometry curves presented an evident bump with increasing potential. This change should be related to numerous nuclei formed on the electrode surface, resulting in the sharp increase in electrode surface area. Along with crystal growth, its surface area will be further enhanced. Instead of crystal growth, nucleation predominated the increase in surface area at this stage. Several crystals may contact and connect with each other with prolonged time. The surface area reduced along with this change. The crystals continually grew after the connection, possibly causing the increase in surface area. Along with the serious consumption of active ions around the electrode surface, the diffusion of active ions gradually controlled the reactions. The interaction among the three factors resulted in the decline in current after approaching a peak value. With further prolongation of time, the reactions will be completely control by active ion diffusion; the current will decline rapidly and approach a constant value. Correspondingly, a flat electrode surface with an approximately stable surface area formed. Evidently, the potential significantly influenced the duration of the third stage. Specifically, the shorter duration resulted from a higher potential. A high potential indicated a stronger driving force for ionic migration and crystal growth, the crystals will connect to each other at a higher rate. As a result, a flat electrode surface will be formed in a shorter duration.

**Figure 4:**
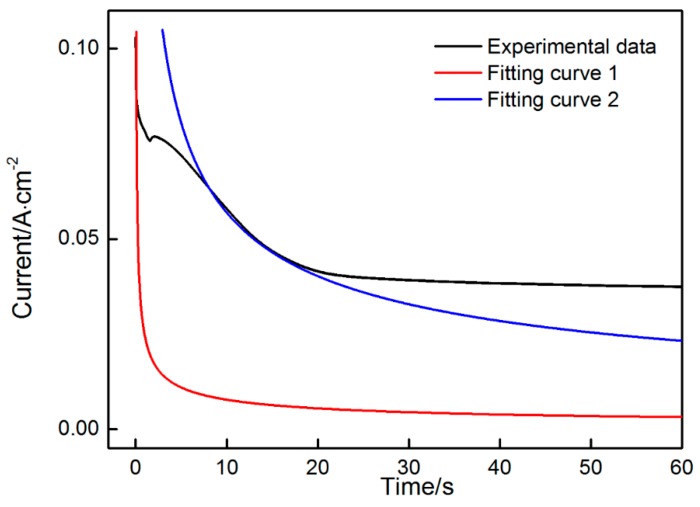
Fitting curves obtained at the first and third stages when a potential of 2.0 V was applied.

The Cottrell equation was also applied to fit the data obtained at the first and fourth stages at the applied potential of 2.0 V. The fitting results are indicated in Figure 4. When the potential was applied, the obtained data in an instant at the first stage can be expressed effectively by the Cottrell equation (Curve 1). However, the subsequent data seriously deviated from the fitting curve due to the incubation of a large number of nuclei on the electrode surface. During MnO_2_ nucleation and growth on nickel foam, the electrode surface area changed dynamically. Fitting was unsuitable due to the uncertainties in the electrode surface area at the second and third stages. However, when a thin layer of MnO_2_ with stable morphology covered the surface of the nickel foam completely, the electrode surface area stabilized. A similar fitting can be carried out at the fourth stage (Curve 2). The obtained data in the initial period (10–20 s) at the fourth stage agreed well with the theoretical data. However, the data obtained after 20 s gradually deviated from the fitting curve (located above the fitting curve). This trend may be related to the poor electric conductivity of MnO_2_. When the electrode surface is covered with MnO_2_, the reaction rate on the electrode surface will be significantly reduced due to the increase in electrical resistance of the electrode. The diffusion rate of active ions can match with the reaction rate and the current showed no decline with time but stabilized. The fitting results are as follows.

At 2.0 V potential:

The first stage shown in Fitting curve 1: $i_{t}=0.02452t^{-1/2},\;\frac{nFAD^{1/2}C}{\pi }=0.02452$

The fourth stage shown in Fitting curve 2: $i_{t}=0.1799t^{-1/2},\;\frac{nFAD^{1/2}C}{\pi }=0.1799$

*F*, *D*, *C* and *n* are all unchanged parameters in the two Cottrell equations. The surface area of electrode (*A*) will change significantly due to MnO_2_ deposition. Therefore, the ration of electrode area at the first and fourth stages approximated 7.34, that is, the surface area of the electrode deposited with MnO_2_ increased approximately six times when compared with that prior to deposition. Thus, a porous material with very high specific area was synthesized by electrodeposition.

The nucleation mechanisms can be revealed by further analyzing the relation between time and current. Nucleation is usually divided into the instantaneous and the progressive process. Instantaneous nucleation indicates the simultaneous formation of a large number of nuclei. On the contrary, nucleation may occur throughout the entire deposition process in progressive nucleation. Hills has derived a model to identify the predominating mechanism during nucleation.

For instantaneous nucleation:

(7) $$i_{\left(t\right)}=\frac{zFN_{o}\pi {\left(2DC\right)}^{3/2}M^{1/2}}{\rho^{1/2}}t^{1/2},$$

For progressive nucleation:

(8) $$i_{\left(t\right)}=\frac{2zFK_{n}N_{o}\pi {\left(2DC\right)}^{3/2}M^{1/2}}{3\rho^{1/2}}t^{3/2},$$

in which *z* denotes the number of electrons transferred in the electrochemical reaction; *N*_0_ represents the number of nuclei initially formed; *D* denotes the diffusion coefficient of active ions (cm^2^/s); *C* refers to the concentration of active ions in the electrolyte (mol/cm^3^); *M* denotes the atomic weight of the deposit (g/mol); *ρ* represents the density of the deposit (g/cm^3^); *K_n_* indicates the constant; *i*_(*t*)_ refers to the current obtained at the time of *t* (*A*); *t* represents the deposition time (s).

**Figure 5:**
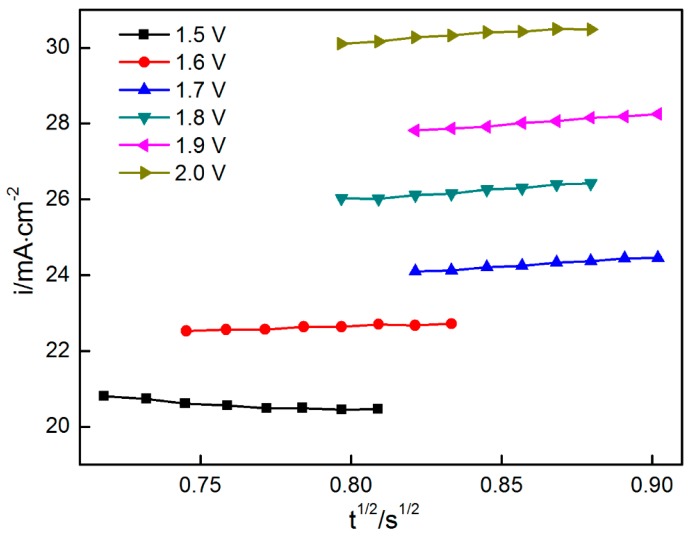
*i*–*t*^1/2^ relationship when the potential was changed from 1.5 V to 2.0 V.

**Figure 6:**
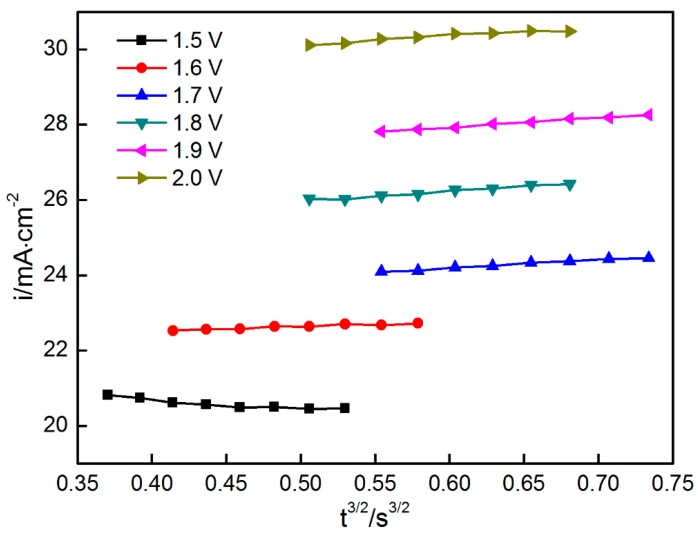
*i*–*t*^3/2^ relationship when the potential was changed from 1.5 V to 2.0 V.

If nucleation is instantaneous, then the current is linear with *t*^1/2^. If nucleation is in progressive mode, then the current forms a linear relationship with *t*^3/2^. The data from the current-rising period of the chronoamperograms, as shown in Figure 3, were collected to establish the relationship between *i* and *t*^1/2^*/t*^3/2^. As shown in Figure 5 and Figure 6, *i* versus *t*^1/2^ and *t*^3/2^, respectively, showed a good linear relationship. The nucleation mechanism cannot be distinguished from the two figures. This finding may be related to the hypothesis in which the other parameters except for the current are unchanged with the time. The hypothesis states that the linear relationship between time and current is established. However, several parameters (such as *D* and *C*) are not constant values.

The Scharifker-Hills (SH) model, by normalizing the current and the time to the peak current and the peak time, proves to be more convincing than Hills model, when can be expressed by the following equations.

For instantaneous nucleation:

(9) $${\left(i/i_{m}\right)}^{2}=\frac{1.9542}{t/t_{m}}{\left\{1-\exp\left[-1.2564\left(t/t_{m}\right)\right]\right\}}^{2},$$

For progressive nucleation:

(10) $${\left(i/i_{m}\right)}^{2}=\frac{1.2254}{t/t_{m}}{\left\{1-\exp\left[-2.3367\left(t/t_{m}\right)\right]\right\}}^{2},$$

**Figure 7:**
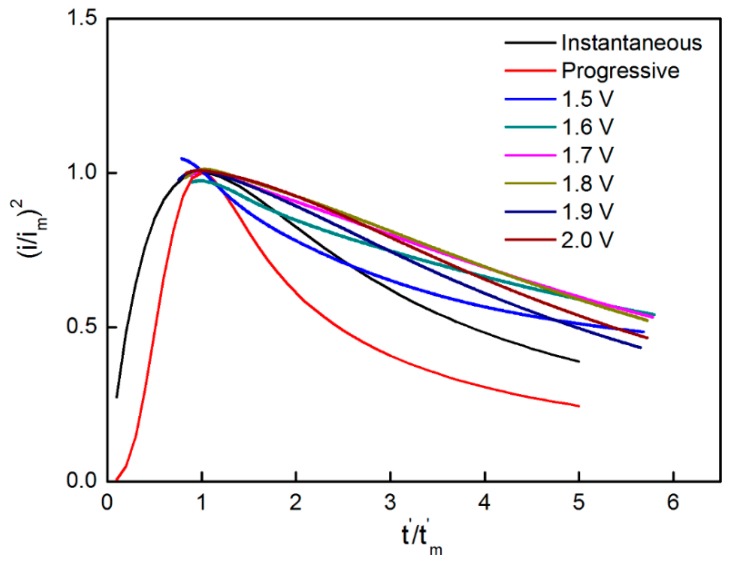
(*i*/*i_m_*)^2^–*t’*/*t’_m_* relationship when the potential was changed from 1.5 V to 2.0 V.

A small delay (*t*_0_) should be deducted to describe the nucleation mechanism more accurately as *t* in the equations represents the time when nucleation starts. Thus, *t* and *t_m_* are represented by $t^{\prime }$ and ${t_{m}}^{\prime }$ ($t^{\prime }=t-t_{0}$, ${t_{m}}^{\prime }=t_{m}-t_{0}$), respectively and the results are shown in Figure 7.

Figure 7 shows the relationship curves of (*i*/*i_m_*)^2^ and *t’*/*t_m_’* under different potentials and the theoretical curves of instantaneous and progressive nucleation obtained from the SH model. The figure shows that the curves obtained from the experimental data agree well with that describing the instantaneous nucleation, thereby indicating that the instantaneous nucleation is predominant over the deposition of MnO_2_ on nickel foam. Tuyen et al. [47] prepared the NixCo1–*x*(OH)_2_ hydroxide films on carbon nanofoam paper by the electrodeposition for high area capacity supercapacitor electrodes. The nucleation and growth mechanism of the films were also investigated in detail by chronoamperometry. The results revealed that the experimental curves between (*i*/*i_m_*)^2^ and *t*’/*t_m_’* deviated from the theoretical curves. Comparatively, the theoretical curves are closer to those instantaneous nucleation mode, showing agreement with our experimental result. Tuyen et al. [48] also synthesized the nickel cobalt hydroxide films on stainless steel by electrodeposition. In order to investigate the nucleation mechanism, the current transients were fitted with three nucleation models, corresponding to Scharifker–Hill, Scharifker–Mostany and Mirkin–Nilov–Heerman–Tarallo. The results demonstrated that a 3D instantaneous nucleation mechanism was predominant over the film growth, which was also confirmed by AFM and FEG-SEM.

### 3.3. Chemical Compositions and Phase Constituents of the Deposit

**Figure 8:**
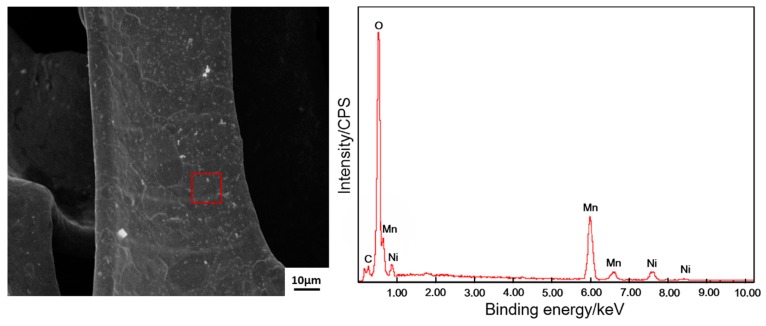
The SEM image of the electrode surface after depositing for 120 s and the EDS result for the selected zone as marked with a red square.

**Figure 9:**
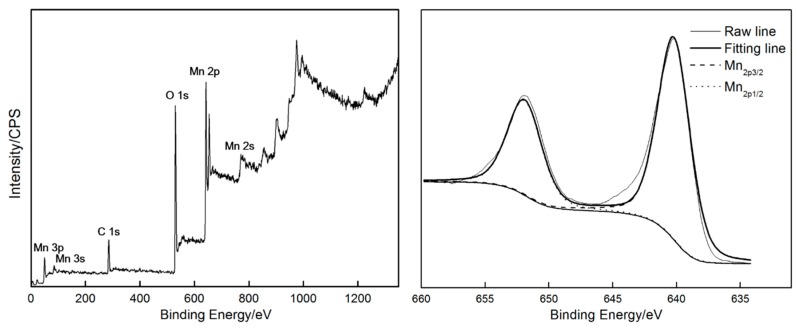
The survey and Mn_2p_ XPS spectrum of the product after depositing for 120 s.

**Figure 10:**
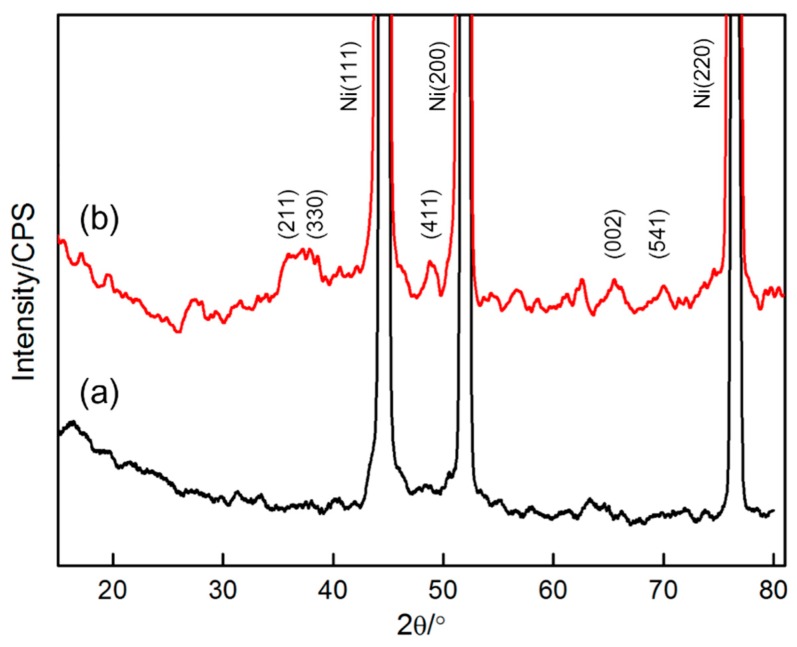
XRD patterns of: (**a**) nickel foam; (**b**) nickel foam covered with the product after depositing for 120 s.

Phase compositions of the deposit was identified by EDS (shown in Figure 8). The selected zone primarily comprised Mn (26.57 at. %), O (28.59 at. %) and Ni (42.86 at. %). Ni originated from the nickel foam substrate. The deposit should be rich in Mn and O. Therefore, the deposit should be a type of oxide-containing manganese. The stoichiometric ratio of Mn and O in the deposit can be inferred through EDS results. There are the comparatively large measure errors for oxygen as a light element, since the characteristic X-ray from oxygen is very low in intensity and it can be strongly absorbed by the beryllium window of Si (Li) detector in EDS. To accurately identify the phase constituents of the deposit, XRD and XPS analyses were carried out (Figure 9 and Figure 10, respectively). The survey spectrum shows that Mn and O were the main elements in the deposit. On the other hand, a narrow spectrum was used to identify the chemical state of the deposit. The spectrum consisted of two strong peaks located at 640.0 and 652.2 eV, respectively, which can be identified as Mn_2p3/2_ and Mn_2p1/2_ peaks. These results indicate that Mn ion exists in the form of Mn^4+^ in Mn oxide deposit, thereby indicating the successful deposition of MnO_2_. Figure 10 shows the XRD patterns of nickel foam without any deposit and nickel foam covered with the product of 120 s deposition. The two patterns exhibited three strong diffraction peaks, which were confirmed to be related to nickel foam (JCPDS card No. 04-0850). When the substrate was deposited for 120 s, several characteristic diffraction peaks belonging to MnO_2_ (JCPDS card No. 044-0141) can be observed, further confirming that the deposit should be MnO_2_.

### 3.4. Morphological Evolution of MnO_2_

**Figure 11:**
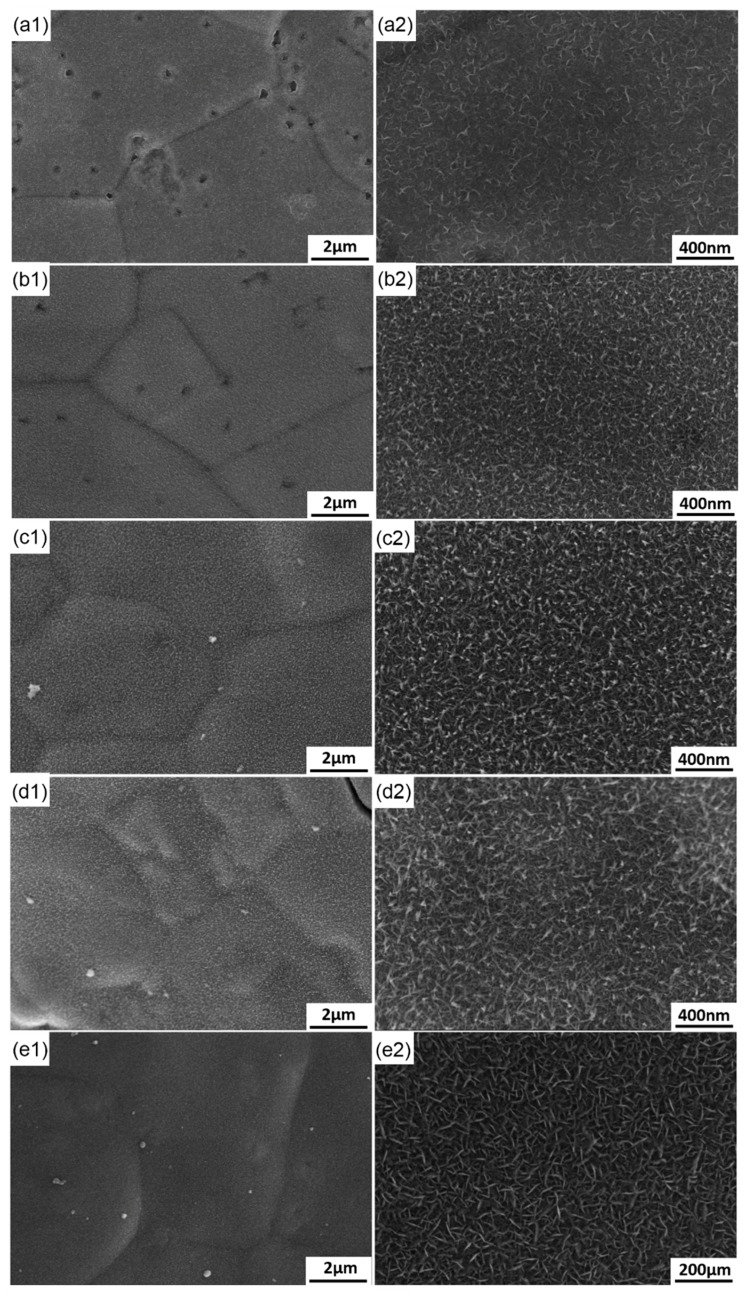
The morphological evolution of the deposits. (**a**) 3 s; (**b**) 7 s; (**c**) 20 s; (**d**) 40 s; (**e**) 120 s.

MnO_2_ was deposited on nickel foam at the potential of 2.0 V, which was applied at different time periods. The morphological evolution of MnO_2_ with the change in deposition time was investigated in detail (shown in Figure 11). As shown in Figure 10a, the initial nickel foam comprised numerous equiaxed grains with an average diameter of approximately 50 nm and grain boundaries can also be observed. The nickel foam surface was comparatively smooth and no impurities were observed. When the potential of 2.0 V was applied for 3 s, the nickel foam surface was covered with a thin layer of substances and showed roughness (Figure 11a). Several fine white whiskers protruded from the surface and connected with each other, resulting in the formation of a network structure. Combined with the results shown in Figure 3, a large number of MnO_2_ nuclei were formed instantaneously, then connected with each other in an extremely short time (less than 3 s). Prior to the connection, nuclear growth was omnidirectional, that is, growth rate was approximately the same in the directions perpendicular and parallel to the sample surface. However, once connection is completed, the grains will grow preferentially in the direction of the applied electric field (perpendicular to the sample surface). Comparatively, the growth rate of the grain boundary was higher than that of the grain itself due to the higher energy in the former. As a result, numerous fine white sheets with a length of about 50 nm grew along grain boundaries and connected to the honeycomb structure. When the deposited time was prolonged to 7 s, the grain boundaries of initial nickel foam became indistinct (Figure 11b), thereby indicating that more MnO_2_ were deposited. Additional fine white sheets grew from the grain boundaries, resulting in the formation of a rougher surface. Thus, the surface area of the electrode increased when deposition time was increased from 3 s to 7 s. However, the increase in surface area caused no corresponding enhancement in the current (shown in Figure 3). The disagreement may be attributed to the change in concentration of active ions around the electrode surface. The formation of numerous nuclei and their subsequent connection consumed the majority of active ions around the electrode surface. Thus, grain growth mainly depended on active ions diffusion. Therefore, as the crystal growth caused the increase in surface area, the current gradually reduced at 3–7 s. With the increase in deposition time to 20 and 40 s, the grain boundaries of initial nickel foam can be discerned. A layer of porous MnO_2_ covered the nickel foam surface completely. Compared with Figure 11b, numerous white equiaxed particles with the diameter of approximately 40 nm formed at certain sites in the honeycomb structure and uniformly distributed in the entire surface. The formation of equiaxed particles is related to the poor conductivity of MnO_2_. With the continuous deposition of MnO_2_, the growth rate of some specific zones protruding into the solution may be significantly reduced due to high electric resistance in these areas. These zones will grow in two-dimensional directions, resulting in the formation of several particles. The equiaxed particles further connected to the fibrous particles with a large size of about 100 nm when the deposition time reached 40 s. When deposition time was further prolonged to 120 s, the electrode surface has been completely covered by MnO_2_. Thus, the grain boundaries of the initial nickel foam cannot be identified. The white network structure MnO_2_ with a high specific area are formed and active ion diffusion completely controlled the deposition of MnO_2_. As a result, the current also stabilized (shown in Figure 3). The porous morphology of uniform and dense MnO_2_ deposit layer is a main reason for the increase in the electrode surface area, which will contribute to the enhancement in specific capacitance of the electrode.

Apart from the applied potential, the other factors that significantly affect deposition in terms of nucleation and growth include the surface microstructure of the substrate, thickness of the deposited substance, instinct of the deposition substance and substrate and concentration of hydrogen ions and active ions in the electrolyte. At the microscopic level, nuclei will form preferentially in the surface zones rich in defects (such as vacancies and boundaries) owing to their high energy. At the macroscopic level, nucleation occurs more easily in the vicinity of the electrode surface (such as edges and corners) due to the edge effect (field line clusters at the edges/corners, resulting in a higher current density when compared with the other zones). Deposition rate is also associated with the thickness of the deposited substance. Given the poor conductivity of MnO_2_, deposition gradually slowed down with increasing thickness of MnO_2_ coating. Along with deposition, the active ions (Mn^2+^) were consumed constantly, resulting in depletion of Mn^2+^ around the electrode surface. The deposition rate of MnO_2_ reduced correspondingly with prolonged time. The concentration of hydrogen ions is (pH value) also an essential factor that affects deposition. On the other hand, MnO_2_ is synthesized primarily by the hydrolysis pathway. Reactions (4) and (5) indicate that high hydrogen ions (low pH value) retarded the deposition of MnO_2_. PH value also plays a major role in electrodeposition since it directly determines the electrostatic interaction between the electrode and the deposit [40]. Nickel foam was constantly covered with a very thin layer of nickel oxide in air. Therefore, MnO_2_ was assumed to be synthesized on the nickel oxide surface. The isoelectric point of nickel oxide is approximately 7.8 [49]. However, it is about 3.6 for MnO_2_ [50], indicating that it is easier for MnO_2_ to precipitate on the electrode made of nickel foam in the solution with pH values ranging from 3.6 to 7.8. PH value was about 3.2 in the initial solution. Accompanied with deposition, the concentration of hydrogen ions gradually enhanced around the electrode surface (pH value was reduced). Accompanied with this, a force hindering the deposition increased. The external force resulting from the applied potential will promote the deposition. Comparatively speaking, the latter is higher than the former. Therefore, MnO_2_ deposits on nickel foam continuously.

### 3.5. Evaluation of Electrochemical Performance

**Figure 12:**
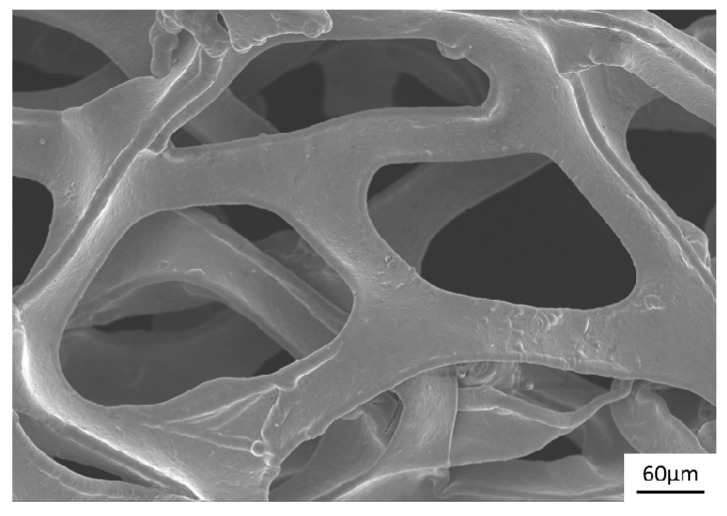
The SEM image of the initial nickel foam surface.

The substrate is essential for the final electrochemical performance. Figure 12 shows the structure of the initial nickel foam. The nickel foam exhibited a loose porous structure, within which a large number of lath-shaped branches were cross-connected in three dimensions. Numerous pores with a diameter of approximately 300 μm can be observed among the network framework. The majority of pores were lined with the outside environment, indicating that nickel foam can carry more active substances as a support due to its very high specific surface area when compared with a dense planar electrode. The test results also confirmed this finding. A planar graphite sheet as an anode was used as substrate for depositing MnO_2_ with a potential of 2 V (vs. SCE) for 60 and 120 s in 0.14 M Mn_2_SO_4_. The mass of deposited MnO_2_ reached 0.600, 1.134 and 1.733 mg, thereby presenting an increasing trend with prolonged deposition time. When the nickel foam sheet was considered a substitute for the graphite sheet, the mass of deposited MnO_2_ increased to 1.067, 2.000 and 2.233 mg under the same experimental conditions. Evidently, the nickel foam carried more MnO_2_ than the graphite with the same external surface area.

**Figure 13:**
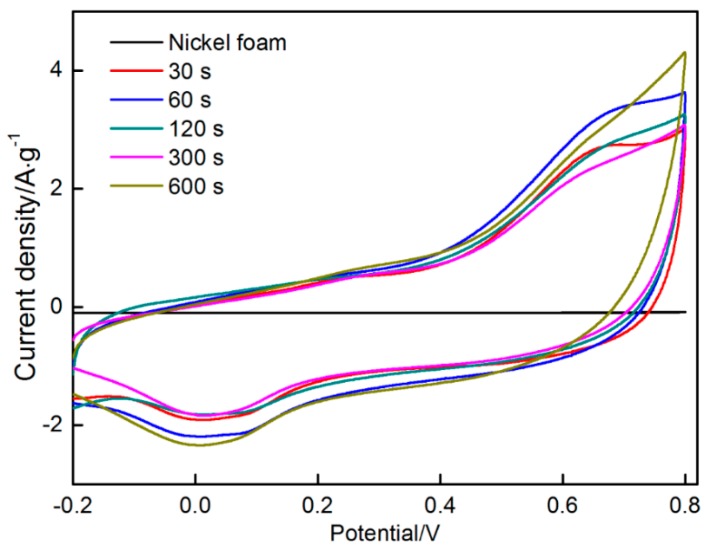
CV curves taken from nickel foam and MnO_2_ deposits.

Figure 13 shows the results of CV of MnO_2_ electrodes deposited on nickel foam at various time (30, 60, 120, 300 and 600 s). The nickel foam without any deposit was selected for testing. The scanning rate of CV test is 0.005 mV/s, whereas the scanning potential is swept from −0.2 V to 0.8 V. The CV curve taken from nickel foam without MnO_2_ deposition is approximately regarded as a line, indicating the integral area surrounded by the curve can be ignored. Thus, specific capacitance of nickel foam can be negligible. Other curves presented a rectangular shape with large integral area, indicating that MnO_2_ exhibited a good capacitive performance. It can be found that the current changed instantaneously with the change in scanning direction, demonstrating the good dynamic reversibility of the charging and discharging processes. The charging and discharging processes can be described by the following equation [42,51].

(11) $${\mathrm{MnO}}_{2}+X^{+}+e^{-}↔{\mathrm{MnOOC}}^{+},$$

in which X^+^ represents the other ions in the electrolyte.

According to the CV curves, the following formula can be applied to calculate the specific capacitance:

(12) $$C=\frac{1}{mv\left(\Delta V\right)}\int_{V_{a}}^{V_{c}}I\left(V\right)dV,$$

in which *C* represents the specific capacitance (F/g), Δ*V* denotes the range of applied potential, *m* refers to the mass of the deposit (g), *v* and *I*(*V*) are the scan rate (V/s) and the current response, respectively.

Specific capacitances of the MnO_2_ electrodes prepared at different time periods (30, 60, 120, 300 and 600 s) measured 220, 242, 226, 206 and 270 F/g, respectively. The electrode at a deposition time of 600 s was higher than other electrodes probably because a more uniform and dense MnO_2_ electrode was obtained at a longer deposition time.

**Figure 14:**
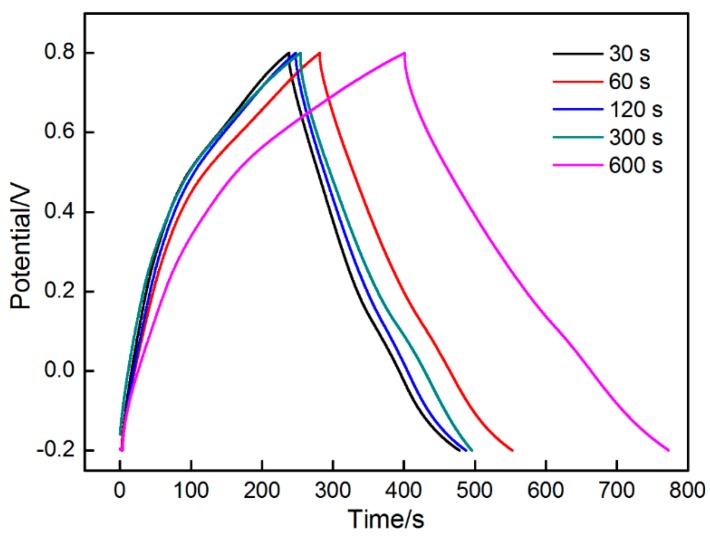
Galvanostatic charge/discharge curves taken from MnO_2_ deposits.

The MnO_2_ electrodes at different deposition time periods were also tested by galvanostatic charge/discharge. As indicated in Figure 14, the profiles exhibited a typical symmetric triangle characteristic, indicating an excellent electrochemical performance obtained in the MnO_2_ electrodes.

Specific capacitance values can also be calculated by equation as follows:

(13) $$C=\frac{I\times \Delta t}{m\times \Delta V},$$

in which *I* represents the current (*A*), *m* stands the mass of the deposit, Δ*V* and Δ*t* indicates the potential window (*V*) and the time (*t*) during cycling, respectively. According to the Equation (13), the specific capacitances of these MnO_2_ electrodes are 250, 279, 261, 265 and 400 F/g.

**Figure 15:**
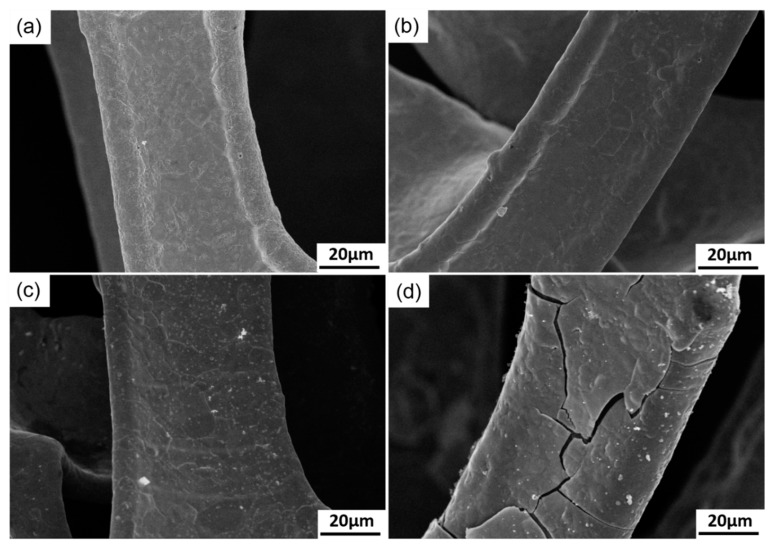
Surface morphologies of MnO_2_ deposited on nickel foam. (**a**) 60 s; (**b**) 120 s; (**c**) 300 s; (**d**) 600 s.

The results indicate that the specific capacitance of MnO_2_ deposited for 30, 60, 120 and 300 s showed no significant statistical difference, which resulted from their very similar morphologies (Figure 15a–c). However, when deposition time was prolonged to 600 s, the specific capacitance of MnO_2_ enhanced by 37.5%. This phenomenon is explained by the effective evolution of MnO_2_ morphology. As shown in Figure 15d, numerous fine gaps were observed in the MnO_2_ coating, resulting from the difference in growth rates in different zones. Slight differences were noted in the micromorphology of different zones. Several zones with high growth rates preferentially protruded into the electrolyte. However, the growth of other zones was restricted. Several tiny gaps formed among the protrusions with different lengths. The active ions were consumed more rapidly in the gaps, whereas those in the electrolyte exhibited difficulty in diffusing into the gaps. The two factors accelerated the micro-gap to macro-gap transition. The development of these fine gaps contributed to the enhancement in specific surface area of the electrode to a certain extent. Thus, these gaps may be the major factors that increase the specific capacitance of MnO_2_ deposited for 600 s. However, these results provide no guarantee that the specific capacitance of MnO_2_ can be improved persistently with prolonged deposition time. The coating thickness of the gap depth increased in a longer deposition time. The coating also increasingly loosened due to the reduction in concentration of active ions in the electrolyte and electrode reaction, which is fully controlled by ion diffusion, thereby resulting in peeling off in specific local zones from the coating. This condition not only reduces the specific capacitance of the electrode but also deteriorates its service stability. Therefore, the suitable deposition time should not exceed 600 s.

## 4. Conclusions

1. As shown by analyzing of chronoamperometry and SEM, the process of deposition of MnO_2_ on nickel foam contained four stages. After an extremely short incubation period (the first stage), the exposed nickel foam was instantly covered by a large number of MnO_2_, then nuclei were connected after a very short time (less than s) at the second stage. With the continuously competitive growth of boundaries and grains, the morphology of MnO_2_ electrode surface changed from fine sheet network structure to honeycomb structure (the third stage). At the fourth stage, the electrodeposition of MnO_2_ was diffusion-controlled, resulting in the formation of porous MnO_2_;
2. MnO_2_ electrode with had a very high specific surface area (about 6 times that before the electrode deposition), which resulted in a high specific capacitance. MnO_2_ coatings deposited for different time (30, 60, 120, 300 s) exhibited a similar specific capacitance (CV: about 224 F/g; galvanostatic charge-discharge: about 264 F/g). Comparatively speaking, the value of MnO_2_ deposited for 600 s was highest (CV: 270 F/g; galvanostatic charge-discharge: 400 F/g).
